# Teaching patient-centered communication skills: a telephone follow-up curriculum for medical students

**DOI:** 10.3402/meo.v19.22522

**Published:** 2014-04-25

**Authors:** George W. Saba, Calvin L. Chou, Jason Satterfield, Arianne Teherani, Karen Hauer, Ann Poncelet, Huiju Carrie Chen

**Affiliations:** 1 Department of Family and Community Medicine, University of California, San Francisco, CA, USA; 2 Department of Medicine, University of California, San Francisco, CA, USA; 3 Veterans Affairs Medical Center, San Francisco, CA, USA; 4 Department of Neurology, University of California, San Francisco, CA, USA; 5 Department of Pediatrics, University of California, San Francisco, CA, USA

**Keywords:** education, medical, undergraduate, clinical clerkship, patient-centered care, physician–patient relationship, continuity of patient care

## Abstract

**Background:**

To encourage medical students’ use of patient-centered skills in core clerkships, we implemented and evaluated a Telephone Follow-up Curriculum focusing on three communication behaviors: tailoring education to patients’ level of understanding, promoting adherence by anticipating obstacles, and ensuring comprehension by having patients repeat the plans.

**Methods:**

The intervention group consisted of two different cohorts of third-year medical students in longitudinal clerkships (*n=*41); traditional clerkship students comprised the comparison group (*n=*185). Intervention students telephoned one to four patients 1 week after seeing them in outpatient clinics or inpatient care to follow up on recommendations. We used surveys, focus groups, and clinical performance examinations to assess student perception, knowledge and skills, and behavior change.

**Results:**

Students found that the curriculum had a positive impact on patient care, although some found the number of calls excessive. Students and faculty reported improvement in students’ understanding of patients’ health behaviors, knowledge of patient education, and attitudes toward telephone follow-up. Few students changed patient education behaviors or called additional patients. Intervention students scored higher in some communication skills on objective assessments.

**Conclusion:**

A patient-centered communication curriculum can improve student knowledge and skills. While some intervention students perceived that they made too many calls, our data suggest that more calls, an increased sense of patient ownership, and role modeling by clerkship faculty may ensure incorporation and application of skills.

Future physicians, regardless of specialty, will need to communicate effectively with their patients. To address this need, medical schools have incorporated teaching of patient-centered communication techniques in the pre-clerkship curriculum (1–7). However, few programs have evaluated the transfer of these skills into patient care settings during the core clinical clerkships (8). Moreover, although core clerkships offer important opportunities for students to practice patient-centered communication skills, they typically provide few continuity experiences to allow students to appreciate the clinical impact of the discussions they have had with patients and families. As a result, students may also miss opportunities to monitor transitions of care or evaluate the success of prescribed interventions intended to prevent recurrence or re-hospitalization. In fact, students express strong negative emotions when witnessing poor care transitions and identify communication gaps that could adversely affect patient care (9). Innovative curricula addressing transitions of care show promise (10), but time and resource constraints during the third year require creative, efficient, and valid training approaches.

We developed a Telephone Follow-up Curriculum (TFC) for third-year medical students (MS3s) to reinforce three specific, measurable patient-centered communication skills. These skills, currently taught in the University of California, San Francisco (UCSF) School of Medicine's pre-clerkship courses, help to tailor the educational message to the patient's level of understanding (‘ask-teach-ask’) (11), promote medical adherence by anticipating obstacles (‘looking ahead’) (12), and ensure patient comprehension of the plan by having the patient repeat the plan back in his or her own words (‘closing the loop’) (13). The purpose of the current study was to explore student and faculty perceptions of the TFC and to evaluate the impact of this reinforcing curriculum on MS3s’ patient-centered communication skills. Our hypothesis was that reinforcing MS3s’ patient-centered communication skills through a TFC would improve these skills in subsequent patient encounters and increase awareness of psychosocial barriers in safe care transitions.

## Methods

### Design and setting

We conducted this prospective, single-institution study of a curricular intervention using both quantitative and qualitative methodology at UCSF and obtained institutional review board approval.

The UCSF pre-clerkship curriculum introduces students to three patient-centered communication skills (‘ask-teach-ask’, ‘looking ahead’, and ‘closing the loop’). The TFC was designed to build upon and reinforce these skills during the core clerkships. In the core clerkship curriculum, students are placed into different clerkship models based on their stated preferences. Models offered include 1) traditional clerkship rotations, 2) 6-month site-based programs where students complete a series of consecutive clerkships at one clinical site, and 3) a 12-month longitudinal integrated clerkship (LIC) (14) where students concurrently complete their core clerkships through discipline-specific preceptorships and continuity with a panel of patients they follow across settings. Students were not aware of the TFC or its implementation in the different curricular models when submitting their clerkship preferences.

### Subjects

Study participants were MS3s on clerkship rotations in 2008–2009 and 2009–2010. In 2008–2009, we implemented the TFC for MS3s in a 6-month site-based program (*Intervention Group 1, or IG-1*). Students in this program, called VALOR, completed their medicine, neurology, psychiatry, and surgery clerkships at a Veterans Affairs Hospital (15). In 2009–2010, we expanded the TFC to include that year's MS3s in VALOR and the LIC (*Intervention Group 2, or IG-2*) (14). In total, 41 MS3s and seven faculty preceptors participated in the TFC during the two intervention years. MS3s completing traditional clerkship rotations (which did not include the TFC) comprised the *comparison groups* (*n=*90 in 2008–2009 *Comparison Group, CG-1; n=*95 in 2009–2010 *Comparison Group, CG-2*).

### Intervention

The objectives of the TFC were to 1) reinforce students’ use of three patient-centered communication skills (‘ask-teach-ask’, ‘looking ahead’, ‘closing the loop’) and 2) deepen students’ understanding of patients’ real-world psychosocial contexts and their obstacles to following action plans. Expected student learning outcomes included increased use of the skills in patient encounters and more attempts to help patients address obstacles to care plan adherence. We also hoped that students would assess patients’ retention and understanding of their care plan from their last medical visits, reinforce clinical recommendations, and address obstacles.

Prior to implementation of the TFC, one author (JS) trained faculty preceptors, explaining their role in the curriculum and ensuring their comfort and proficiency with the three patient-centered communication skills; JS also introduced these skills to intervention group students (IG-1 & IG-2) and oriented them to the expected curricular activities (Appendices 1 and 2). All intervention students attended seminars (2 hours with scripted role plays) and made one to four calls to patients with individual and small group debriefs with preceptors.

IG-1 students selected four patients that they had seen in outpatient clinics or had discharged from inpatient care; they telephoned the patients at home approximately 1 week after their last encounter. Students followed up on clinical recommendations such as medication changes and referrals (Appendix 2), documented the telephone contact in the patient's medical record, and completed a written Post Telephone Call Exercise (PTCE; Appendix 1) after each telephone encounter. Based on IG-1 student feedback, we modified the curriculum to decrease the number of PTCEs required for IG-2 students, who were required to complete one PTCE sometime during the curriculum instead of after every telephone call. Students reviewed their completed PTCEs with faculty preceptors trained in the TFC.

### Evaluation measures

We focused on evaluating students’ knowledge and skills, faculty and students’ perceptions, and change in student behavior or application of knowledge and skills.

#### Survey

The IG-1 students completed a 15-item post-course survey assessing the usefulness and quality of the TFC and self-reported changes in their attitudes, knowledge, and skills regarding doctor–patient communication. Closed-ended questions used a five-point Likert scale (1=strongly disagree, 5=strongly agree). Faculty who worked with IG-1 students completed a 15-item post-course survey that combined Likert scale and open-ended questions to inquire about their impressions of student learning. Based on IG-1 student comments regarding the evaluation process, we streamlined evaluations to obtain focused verbal feedback from IG-2 students regarding the usefulness for learning and quality of the TFC.

#### Focus groups

IG-1 students and their faculty participated in separate focus groups to share their experiences of participating in the TFC. Facilitators who were not involved in teaching the curriculum conducted the separate 1-hour focus groups with both students and faculty.

#### Standardized patient examination

To evaluate students’ ability to apply skills that the TFC reinforced, we constructed a chronic illness case that provided several opportunities for students to demonstrate the three patient-centered communication skills. The case involved a 68-year-old man with a history of congestive heart failure and depression who presented with fatigue and an interest in reestablishing primary care. We included this case as one of three standardized patient (SP) cases in an existing clinical performance examination required for all MS3s. Following standard protocol for all SP examinations at our institution, the SPs received extensive training on all cases used in the exam, including case portrayal, checklist scoring, and exercises to establish inter-rater reliability. For all students, SPs completed a 34-item checklist on history-taking, physical examination, and communication items after each student encounter. SPs did not know which students participated in which clerkship programs and were not aware of the TFC. We compared the performance of students between the intervention and comparison groups on seven communication questions that specifically related to patient education skills (Table 3).

### Quantitative data analysis

#### Satisfaction surveys

We compiled intervention student and faculty evaluations of the curriculum and calculated mean scores for survey data.

#### Clinical performance data

We examined students’ performance during the SP case and compared scores between *intervention* and *comparison* group students using a one-way analysis of variance (ANOVA). Multiple regression analyses were conducted to predict the type (*intervention* vs. *comparison* group) and intensity of the curricular experience (among those who made one, two, and four calls) on clinical performance (SP case).

### Qualitative data analysis

We analyzed the focus group comments using open coding (16) to examine the qualitative data from the PTCEs. Four authors (GS, CC, AT, HCC) read seven PTCEs together to identify initial descriptive codes. Upon reaching agreement, three authors (GS, CC, HCC) reviewed the remaining PTCEs. Using the constant comparative method, we used multi-staged coding, beginning with open coding of raw data to develop key ideas. Axial coding then organized these categories into patterns, and finally, we used selective coding to develop theoretical formulations that linked key variables to themes. Discussion among investigators resolved analytic discrepancies.

## Results

In 2008–2009, 18 students received TFC (IG-1), and 90 students comprised the comparison group (CG-1). Seventeen of the intervention students completed four PTCEs, and one student completed three. During 2009–2010, 33 students received TFC (IG-2), and 95 students constituted the comparison group (CG-2). Although IG-2 students were required to complete one PTCE, 1 student completed four, 7 completed two, and 16 completed one. Nine students’ PTCEs were completed but misplaced. Students did not provide PTCEs for any additional calls they made. Over the 2 years of the study, a total of 105 PTCEs were submitted. Students reviewed their telephone follow-up exercises with their TFC faculty who included five internists and two psychiatrists with 1–16 years of experience as preceptors.

### Characteristics of telephone calls

On PTCEs, students most frequently cited that their goals during the call were to check in about prescribed medications, follow-up appointments, and treatment plans discussed in their last encounter (Table 1). Most of the reported telephone calls did not result in a change in medication or treatment. Students reported accurate patient comprehension and retention of information from their last contact in the majority of their calls. In half of the exercises, students reported good patient adherence to their management plan. In patients with non-adherence, students ascribed the problem to patients’ poor understanding of the plan, situational or structural barriers, or the existence of co-morbid conditions. The majority of exercises revealed that students were surprised by unanticipated information elicited in their conversations with patients or caretakers during the calls (e.g., reasons for non-adherence, level of family involvement in care).

**Table 1:** Characteristics of students’ post telephone call exercises

	No. of responses	%
I. Students’ goals for the call (*n*=92*; some participants identified more than one*)		
a) Check in about:		
Medications (e.g., obtaining them, side effects)	46	50
Follow-up appointments (e.g., recalling the appointment; barriers to attendance)	38	41
Treatment plans	34	37
Physical symptoms	26	28
Understanding of illness	21	23
Emotional state	13	14
Lifestyle modification	10	11
b) Provide subsequent information (e.g., lab results)	7	8
II. Student provided intervention during the call (*n*=69)		
Yes	24	35
No	45	65
III. Students’ assessment of patient comprehension of last contact, e.g., information, plan (*n*=91)		
Yes, patient did comprehend	58	64
No, patient did not comprehend	28	31
Patient comprehended some but not all of last contact	5	5
IV. Students’ assessment of patient retention of last contact, e.g., information, plan (*n*=94)		
Yes, patient did retain	63	67
No, patient did not retain	21	22
Patient retained some but not all of last contact	10	11
V. Students’ assessment of patient adherence to plan from last contact (*n*=102)		
Yes, patient did adhere	51	50
No, patient did not adhere	39	38
Patient adhered to some but not all of plan from last contact	12	12
VI. Students’ assessment of reasons for patient non-adherence to plan from last contact (*n*=48)		
Did not understand	15	31
Barriers to adherence	15	31
Co-morbidity	13	27
Other (e.g., did not remember; chose not to adhere)	5	11
VII. Content of call surprised students with unanticipated information (*n*=80)		
Yes	54	68
No	26	32
VIII. Learning points from the telephone follow-up call exercise		
a) Techniques focused on in this curriculum (*n*=55; *some participants identified more than one*)		
Ask teach ask	25	45
Closing the loop	29	53
Looking ahead	23	42
b) Other techniques (*n*=72; *some participants identified more than one*)		
Write down a plan for the patient at the time	19	26
Use phone calls to motivate patients and foster behavior change	13	18
Involve family or caretaker	8	11
Make simple plans for patients	7	10
Have patient education materials available at visit (e.g., handouts, websites)	7	10
Decrease number of medications	2	3
Other	23	32

Total combined *N*=105.

### Perceptions of the curricular experience

Nearly all PTCEs (96%) contained student comments about the value of the follow-up exercise (Table 2). Positive comments reflected students’ appreciation for follow-up information on their patients and opportunities to make additional interventions.

Doing the phone follow-up gave me a glimpse into what happens when patients leave the office and put the treatments prescribed in the office into action.

[I] was able to remind [the] patient of something he had forgotten … he did not remember that he had been told to eat a low-salt diet. Also had not yet made his follow-up appointment and appreciated the reminder.

**Table 2:** Students’ assessment of the value of the telephone follow-up call

Students’ commentary about value of the telephone follow-up call (*n=*101)	85
Positive	85
Negative	4
Students noted both negative and positive comments	12
Reasons students gave for rating of value of the telephone follow-up call (*n=*99; *some identified more than one*)	
Positive value	
Liked knowing what happened	29
Patient appreciation	27
Ability to make an intervention	24
Deepened relationship-partnering with patient in care	18
Practice skills	14
Curricular requirement ensured learning of skills	3
Negative value	
Timing of calls in clerkship was too late	4
Logistics of call	4
Other	16

Students also noted how the calls led to a deepening of the relationship with the patient and that the patients expressed appreciation for the contact.

It really gave me a sense that I was in an ongoing partnership with the patient, and I think she appreciated the call as well.

Finally, students valued the opportunities to practice and ensure learning of the relevant communication skills.

Reflecting on the phone interview allowed me to think about the process of delivering information and in doing this, I realize that I am not as explicit with the communication techniques as I would like to be.

Some students discussed how deciding to telephone more than the required number of patients depended on their sense of responsibility.

I was more likely to call if I knew they were coming back at some point-it's really my patient.

I just didn't feel ownership of patients to the extent it seemed useful and even appropriate to call in a lot of cases.

Negative student comments focused on the requirements of the curriculum (preferred the calls to be elective, too many required calls) and logistical challenges of making telephone calls.

It's a reasonable learning activity … it is just difficult to schedule times to write everything up with so much other learning taking place throughout the week.

LIC students, in particular, recommended that the PTCE occur early in the year when they are beginning to engage their patient panels.

### Changes in attitudes, knowledge, and skills

On the post-course survey, the majority of students agreed or strongly agreed that they learned about patient health behaviors (77%), that their ability to provide patient education improved (71%), and that these communication skills will be relevant to future patient encounters (94%). However, only 41% of students reported having made changes to what they do during patient visits because of the TFC.

All five faculty preceptors for the IG-1 students agreed or strongly agreed on the post-course survey that the telephone follow-up calls were a valuable learning experience for students. Faculty preceptors believed that the exercise highlighted the gap between what clinicians think they have said and what patients understand, and that students learned even a single call could have significant impact on patient care. As one faculty member said, ‘I thought it effectively illustrated how fragile a treatment plan can be and how powerful a telephone call [can] be in reinforcing a treatment plan’.

Several faculty hypothesized why students found the curriculum challenging. Some felt that students may have perceived less learning from these exercises because they ‘did not seem to fully appreciate the impact they made’. A related theme concerned role modeling, with another faculty stating, ‘Students don't see attendings and housestaff making follow-up calls’.

### Transfer of learning and ability to apply new skills and changes in behavior

Data from PTCEs showed that a significant number of patients did not comprehend (31%) or retain (22%) information from their last clinical contact and were not adherent (38%) or did not understand (31%) their treatment plans. In response to these findings during their telephone follow-up calls, a number of students (35%) provided interventions during their calls to improve individual patients’ care.

Of the seven communication outcome variables examined in the SP exercise, IG-1 students scored higher than the comparison group CG-1 on three techniques, and IG-2 students scored higher than CG-2 students on a different technique (Table 3). We found no differences between intervention and comparison groups when we separately compared the performance of the VALOR and LIC students in IG-2 with CG-2; combined IG-1 and IG-2 into one intervention group with the one comparison group (CG-1 + CG-2) in a linear regression; or used regression analyses to look at performance of the IG-2 students in relation to the number of PTCEs completed.

**Table 3:** Comparison of curriculum participants vs. non-participants performance on pertinent follow-up items from the standardized patient exam

		2008–2009			2009–2010		
			
Technique	Question	Participants (IG-1) *N=*17	Non-participants (CG-1) *N=*90	*p*	Participants (IG-2) *N=*34	Non-participants (CG-2) *N=*95	*p*
Ask-teach-ask	Asked for my opinion or if I had concerns about my illness	29.4*	37.8*	NS	44.1*	25.3*	0.03
Ask-teach-ask	Warned me about dangers of hypertension	41.2	14.4	0.009	32.4	32.6	NS
Ask-teach-ask	After providing information, asked if I had further questions	17.6	3.3	.02	20.6	15.8	NS
Looking ahead	Helped me identify ways to check my blood pressure	11.8	7.8	NS	–	–	NS
Looking ahead	Helped me map out action plan for diet and/or medication adherence	94.1	84.4	NS	91.1	88.0	NS
Looking ahead	Recommended visit with dietitian or nutritionist	0	12.2	NS	15.1	18.0	NS
Closing-the-loop	Asked me to repeat back in my own words the action plan	29.4	11.1	.05	20.6	10.5	NS

* Numbers represent percentages of students that addressed item correctly on the standardized patient exam.

## Discussion

Our evaluation of the TFC yielded encouraging but mixed results which may be best understood through the framework of the first three levels of Kirkpatrick's model of evaluation, modified for medical education (17–19):

1. *Level 1-Perceptions of the curricular experience:* Students enjoyed the curriculum overall and saw the value of TFC for their personal learning and for their patients’ care.
2. *Level 2-Changes in attitudes, knowledge, and skills:* Calls allowed students to assess their patients’ comprehension, retention, and adherence after in-person encounters – important clinical feedback data typically not available to students. Both students and faculty reported improvement in students’ understanding of patients’ health behaviors, knowledge of and skills in communication related to patient education, and attitudes toward telephone follow-up in patient care.
3. *Level 3-Ability to apply new skills and changes in behavior:* Few students reported changing their patient education behaviors. In addition, few made telephone follow-up calls to additional patients beyond the number required, despite stating that the calls improved patient care. Finally, in observed skills assessments up to 4–5 months after the curricular intervention, the intervention students only demonstrated improvement in three of seven (IG-1) or one of seven (IG-2) communication outcome variables.

Combining data from both years, no significant difference was found in the communication behaviors of the intervention and comparison groups. Interestingly, IG-1 students, who each completed 4 PTCEs, performed better than IG-2 students, who completed fewer PTCEs. It is possible that we might have seen a difference had we required IG-2 students to complete more PTCEs. Students may not always know or want what is best for their learning (20), and our modifications to TFC to improve satisfaction may have resulted in negative learning outcomes.

Another explanation for the different findings over 2 years might be a change in the comparison group students from one academic year to another. The CG-2 students improved in two of the parameters that had shown significant differences between CG-1 and IG-1 students. There were no other curricular changes to explain the comparison group's skill improvements. Although it is possible that elements of the TFC could have generalized to non-participants, the etiology for this baseline improvement is unclear and deserves further study.

It is interesting that, despite self-reported gains in knowledge and skills and perceived improvements to their patients’ care, few students made telephone follow-up calls to additional patients to further their communication skills. A number of students acknowledged that they did not telephone additional patients because they did not feel ownership over those patients’ care. This sense may have limited their opportunities to practice communication skills and cement further learning. Yet, the TFC provides a tangible way for MS3s to assume authentic roles in reducing communication gaps that adversely affect patient care (9). It is possible that students may develop improved communication skills by further promoting continuous patient relationships in their core clerkships, thereby increasing their sense of accountability and responsibility for patient care (21–25).

Another factor in students not making additional calls may relate to a lack of modeling. Focus group faculty mentioned that students do not observe residents or faculty calling patients in between visits. In addition, telephone calls to patients are not a routine expectation of clerkships and students are not formally evaluated on them. These factors may result in a hidden curriculum (26) that suggests to students telephone follow-up calls are not a typical or essential part of real practice. To ensure that students do not view certain activities as curricular busy-work and that skills learned in the classroom are reinforced during the clerkship experience, modeling from clinical supervisors is critical.

Although students demonstrated no measurable change in their behavior with subsequent patients or in a formal assessment, students seemed to have positive effects on patient care while participating in the TFC. In their telephone follow-up calls, students made a wide range of interventions in response to significant rates of poor patient retention, comprehension, and adherence to treatment plans. The TFC resulted in immediate benefits to patients and improved quality of care, and this arguably supports further implementation of this curriculum. Perhaps evidence of tangible effects on improved patient care can be leveraged to change the hidden curriculum, improve student engagement in the curriculum, and increase student practice of telephone follow-up.

## Limitations

We introduced TFC into only one medical school, although the clerkships occurred at multiple private and public hospital and clinic settings. While the demographics of the intervention and comparison group students do not differ (14, 15), it is unclear if one group valued patient-centered communication skills more than the comparison group. Presumably, students electing these longitudinal programs may hold increased interest in continuity of care, thereby introducing selection bias. There were non-equal numbers of data points in each of the 2 years of the curriculum. The IG-1 students made four phone calls and completed four PTCEs each, whereas IG-2 students completed a minimum of one phone call (based on the required one PTCE). This change in the degree of experience did not permit a direct comparison between the samples of the 2 years, although it does suggest that more experience with phone calls may improve skills to a greater degree. Future research would benefit from directly ascertaining patients’ experiences of their follow-up telephone encounters with students.

One important challenge in medical education is to change learners’ behavior for the benefit of their patients. This study demonstrates that a patient education curriculum can improve student knowledge and skill acquisition, and that more intense or different interventions may be needed to affect student behavior change. While students perceived the number of telephone calls required to be excessive, objective results suggest that more calls may be necessary to ensure incorporation and application of skills. Though we focused on improving the outcomes of our students’ future patients as the ultimate curricular goal, this study suggests that this curricular intervention may also have improved the quality and safety outcomes of their current patients.

## Appendix 1

Post telephone call exercise form

Clerkship Telephone Follow-up

(To be handed in, do not include any patient identifiers)

Student Name:

Date of phone contact:       Duration of call: (minutes)

1. What were your goals for the telephone call? Did you achieve your goals?
2. How, if at all, were patient *retention* and *comprehension* of the medical issues and treatment plan different than what you expected? What were the surprises?
3. How, if at all, was patient *adherence* to the treatment plan different than what you expected? What were the unanticipated issues?
4. Check the strategies used in LAST OFFICE VISIT to improve retention, comprehension, and adherence:
  - Ask-teach-ask (Before launching into a medical mini-lecture, first ask patients about their understanding of the issue, then teach based on their level of understanding, and then ask necessary follow-up questions to deepen or check understanding.)
  - Closing the loop (At the end of the visit, check patient comprehension by saying something like, ‘I want to make sure I explained our plan well enough. Can you repeat back what you are going to do between now and the next visit?’)
  - Looking ahead (After sharing the treatment plan, help the patient anticipate and prepare for obstacles to adherence, ‘What might get in the way of filling your prescription?’)
  - List other strategies used:
5. Check the strategies used in the TELEPHONE CALL to improve retention, comprehension, and adherence:
  - Ask-teach-ask
  - Closing the loop
  - Looking ahead
  - List other strategies used:
6. How will this telephone experience affect the way you run future clinic visits? What might you do differently?
7. What were the best things about this telephone f/u experience? Ways it could have been a better learning activity?
  1=strongly disagree; 2=disagree; 3=neutral; 4=agree; 5=strongly agree
8. My Doctoring Course experiences (small group, preceptorships, etc.) helped me care for this patient   1   2   3   4   5
9. This telephone f/u was a valuable learning experience   1   2   3   4   5
10. I will be more likely to use Ask-Teach-Ask, Closing the loop, and/or Looking Ahead in the future.   1   2   3   4   5
11. We should do more telephone f/u encounters with patients   1   2   3   4   5

Other comments?

## Appendix

**Table:** Appendix 2. Suggested student strategies for enacting techniques during telephone follow-ups with patients

ASK-TEACH-ASK: Check patient retention and comprehension	ASK: ‘We covered a lot in your last appointment. What are the major things that you remember from that visit?’
	‘I'm sure it is hard to remember all the details of the plan we came up with but can you recall the major things you were going to do before your next appointment?’
	TEACH: Check patient retention with the actual plan recorded from the last visit. Provide additional or corrective information or education as necessary.
	ASK: ‘Do you have any other questions about XYZ? What other information would you like to learn about?’
LOOKING AHEAD: Reinforce and encourage	If patient has already followed through, provide reinforcement and encouragement. Help them to problem solve or anticipate any obstacles if appropriate: ‘That's terrific you've already set up that referral appointment. Do you foresee any difficulty in making it to that appointment? Do you have any worries about it?’
	If patient has not followed through, explore why.
	They made need a review of the step-by-step process (e.g., ‘You need to call this number to schedule an appointment for your MRI’).
	They might also need to be reminded of the importance and rationale (e.g., ‘Given that you've already had a stroke, we are really worried that your high blood pressure places you at greater risk for another one. This new medication will help you get your BP under control …’)
	They may lack confidence to carry out the plan. ‘On a scale of 1–10, how confident are you that you will be able to …. You gave yourself a 4. Why not a 3 or 2? What would raise you to a 5 or 6?’ Have patient recall past successes or success of family/friends to build confidence.
	There could be environmental obstacles not discussed in the last visit (e.g., transportation, drug is not on the formulary).
	Patient may hold an alternate model of why s/he is ill and have different ideas about indicated treatment – e.g., substance abuse is a spiritual failing and is best treated by solitary prayer. Try something like, ‘Can you help me understand your thoughts about your illness? What do you think caused it? What treatments do you think will work?’
‘CLOSING THE LOOP’ Check patient comprehension of phone conversation	At the end of the call say something like, ‘I want to make sure I was able to share this information effectively. Could you repeat back what it is you (or each of us) will do before your next appointment?’
	Thank the patient and remind them of their next appointment
